# Screening regional management options for their impact on climate resilience: an approach and case study in the Venen-Vechtstreek wetlands in the Netherlands

**DOI:** 10.1186/s40064-016-2408-x

**Published:** 2016-06-17

**Authors:** J. A. Wardekker, D. Wildschut, S. Stemberger, J. P. van der Sluijs

**Affiliations:** Copernicus Institute of Sustainable Development, Utrecht University, PO Box 80115, 3508 TC Utrecht, The Netherlands; Coöperatieve Universiteit Amersfoort, Geldersestraat 6, 3812 PP Amersfoort, The Netherlands; Department of Environmental Sciences, Statistics and Informatics, Ca’Foscari University of Venice, 30123 Venice, Italy; Centre for the Study of the Sciences and the Humanities & Department of Chemistry, University of Bergen, PO Box 7805, 5020 Bergen, Norway

**Keywords:** Resilience, Rural areas, Freshwater, Climate change adaptation

## Abstract

Freshwater systems provide various resources and services. These are often vulnerable to climate change and other pressures. Therefore, enhancing resilience to climate change is important for their long term viability. This paper explores how management options can be evaluated on their resilience implications. The approach included five steps: (1) characterizing the system, (2) characterizing the impacts of climate change and other disturbances, (3) inventorying management options, (4) assessing the impacts of these on climate resilience, and (5) follow-up analysis. For the resilience assessment, we used a set of ‘resilience principles’: homeostasis, omnivory, high flux, flatness, buffering, and redundancy. We applied the approach in a case study in a Dutch wetlands region. Many options in the region’s management plan contribute to resilience, however, the plan underutilised several principles, particularly flatness, but also redundancy and omnivory for agriculture, and high flux for nature. Co-benefits was identified as an important additional criterion to obtain support for adaptation from local stakeholders, such as farmers. The approach provided a relatively quick and participatory way to screen options. It allowed us to consider multiple impacts and sectors, multiple dimensions of resilience, and stakeholder perspectives. The results can be used to identify gaps or pitfalls, and set priorities for follow-up analyses.

**Electronic supplementary material:**

The online version of this article (doi:10.1186/s40064-016-2408-x) contains supplementary material, which is available to authorized users.

## Background

In the coming decades, climate change will pose considerable challenges to natural and human systems and their management. Freshwater systems provide many resources and services and need to be adapted to (rapid) changes in abiotic circumstances. These include rising air and water temperatures, sea level rise, changes in precipitation patterns, and changes in river discharge, which in turn impact water quality, nutrient and pollutant loads, sediment distribution, drought and flooding regimes, species distribution and growing seasons, and a variety of other aspects (e.g. Kundzewicz et al. 2008; Oude Essink et al. 2010; Woodward et al. 2010; IPCC 2014). Climate change impacts add up to (and interact with) the considerable challenges that such systems already face due to other anthropogenic pressures, such as habitat fragmentation and degradation, ground- and surface water contamination, air pollution, soil pollution, and biodiversity loss (Woodward et al. 2010; IPCC 2014). Policymakers and nature managers face the challenging task of climate-proofing their management strategies to warrant long-term viability of freshwater systems.

The assessment of future impacts of climate change, however, is plagued by large and often irreducible uncertainties. Uncertainties are present in the projections of global average temperature, and more so in its translation into regional climate change, and local impacts and responses. Various approaches exist to deal with uncertainties in climate adaptation (for freshwater examples, see e.g. Groot et al. 2014; Thissen et al. 2015). Two groups can be discerned: top-down and bottom-up approaches (Dessai and Van der Sluijs 2007; Wardekker 2011). Top-down strategies (prediction-oriented) aim to analyse the range of potential changes as accurately as possible, and optimize the impacted system to best meet future climate. Bottom-up strategies (system-oriented) focus on assessing the system’s vulnerabilities, and propose measures that will enhance its ability to cope with future disturbances.

One such bottom-up approach is to enhance the resilience of the impacted system. The concept of resilience emerged from ecology in the 1960–1970’s (Holling 1973; Folke 2006). It was used in relation to the stability of ecosystems and the capacity of a system to recover following some shock or disturbance, without losing its characteristics. Resilience has since been adopted by numerous disciplines, ranging from ecology to psychology, engineering, and disaster studies. Recently, it has also been applied in the context of climate change adaptation. For instance, resilient development has become a central concept in IPCC’s Fifth Assessment Report (IPCC 2014). The definitions of what resilience entails vary from narrow to broad (Adger 2000; Carpenter et al. 2001). Ecological resilience, for example, deals with withstanding shocks, counteracting damage, unpredictability, thresholds of system collapse, and persistence and change. Social-ecological resilience is broader, dealing with adaptive systems, the interplay between disturbance and reorganization, self-organization, and learning. Walker et al. (2004) define it as: “the capacity of a system to absorb disturbance and reorganize while undergoing change so as to still retain essentially the same function, structure, identity, and feedbacks”.

In this paper, we explore how regional management options can be evaluated on their implications for (social-ecological) resilience to climate change. We develop a five-stage approach that can be used to perform a rapid scan of management plans. To illustrate the approach, we apply it in a case study on the peat grasslands of the Dutch Venen-Vechtstreek region. This wetland system, centuries old, has both important societal and ecological functions, and constitutes a typical Dutch landscape that has unique cultural heritage values, however is vulnerable to climate change.

## Approach and methods

Regions within any specific country are faced with numerous trends and management goals. Regional policymakers develop plans and options to cope with these and develop into the future. Such plans and options can impact the resilience of a region to climate change, regardless of whether climate change was specifically taken into account. We present an approach to scanning plans and options for such impacts that can be applied relatively easily in a regional decision-setting, by means of workshops and/or a survey involving decision-makers, experts, and stakeholders. The approach aims to perform a quick scan of the resilience implications, which can point to potential weaknesses or unintended consequences of the plans. These can then be subjected to more in-depth study.

**Fig. 1:**
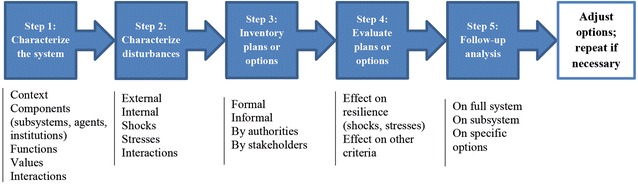
Steps in assessing the effects of regional management options on regional resilience, with examples of potential components/aspects included per step

A wide variety of studies have examined resilience for various systems and pressures (e.g. Adger 2000; Barnett 2001; Carpenter et al. 2001; Walker et al. 2004; Folke, 2006; Dessai and Van der Sluijs 2007; Resilience Alliance 2007, 2010; Wardekker et al. 2010), including climate change. Our approach draws heavily on this large body of earlier work, particularly on the ‘Workbook for Practitioners’ developed by the Resilience Alliance (2010) (step 1–2) and on studies using resilience principles for climate adaptation, such as Barnett (2001) and Wardekker et al. (2010) (step 3–4). Five distinct steps are taken in the analysis (see also Fig. 1):

1. Characterization of the system under study, in terms of key characteristics and functions,
2. Characterization of the issue(s) to which the system should be resilient, in terms of key disturbances,
3. Inventory of (planned or potential) management options for the region,
4. Assessment of the implications of these options, using resilience principles,
5. Follow-up or supplementary analysis.

### Step 1: Characterization of the system under study

Before assessing the resilience implications of any management options, a first step has to focus on establishing what resilience means in the context of the study area: what exactly should be resilient (cf. Carpenter et al. 2001)? Natural and human systems alike are subject to variability, change and evolution, and maintaining the system in exactly the same state or configuration may not be feasible or desirable for a regional management plan. Rather, classic studies of resilience focus on multiple system states or regimes. Various disturbances may have impacts on a system, but if resilience is reduced, they may push the system over a critical threshold, leading to collapse (regime shift) from one state to another (e.g. Folke et al. 2005; Scheffer et al. 2001; Walker and Meyers 2004). Different states are distinguished in the sense that within each state, certain key attributes (function, structure, identity, feedbacks) remain “essentially the same” (Walker et al. 2004). Therefore, an important question is: what characterizes the system under study and its state; what key attributes make one state differ from other potential states? These key attributes define the system and its current or desired state: if they are not retained under climate change, the system has shifted into another state, and consequently, is not climate resilient. For large, complex systems with numerous components and actors, distinguishing among potential states is however not straightforward. Defining the system in terms of key characteristics (what is unique about it?) and key functions (what do we value?) helps to clarify the system and its state.

Characterization starts by providing an overview of the system and its history, as any major changes it underwent over time, thus providing an overview of current and past challenges, the way it is used by various stakeholders, and what stakeholders value about the region. The next step is the definition of key functions of the area. These are the main targets that will need to be protected under climate change. The concept of ‘key functions’ is similar to that of ‘ecosystem services’ (e.g. Millennium Ecosystem Assessment 2005); see the “Discussion”. The key functions are the main desirable aspects of the system which should be made resilient. The climate resilience implications of management options is assessed in relation to these. Determining what is a key function is inherently subjective; it depends on what local stakeholders and decision makers value as the most important aspects of the region/system. Consequently, this step should be participatory or at least based on existing, stated priorities by local actors, using for example existing policy plans, interviews, workshops, and/or other input by stakeholders.

### Step 2: Characterization of the issue(s) to which the system should be resilient

The second step is to answer the question of ‘resilience to what?’ (cf. Carpenter et al. 2001). A distinction can be made between general resilience (to anything) and specified resilience (to specific pressures) (Resilience Alliance 2010). A characterization will need to be made of the key disturbances and their impacts in the system and potential changes in these that are relevant for the management of the area. Disturbances could entail short-term shocks (e.g. natural disasters), ranging from single events to patterns of shocks over time and space (disturbance regimes; e.g. Reice et al. 1990; Nakamura et al. 2000), and long-term pressures (e.g. acidification, increasing temperature, etc.). It is important to retain that disturbances and their impacts in the region under study can also be related to events at a higher system level (e.g. national), a lower system level (specific subsystems, locations, and/or processes in these), or adjacent systems (e.g. a neighbouring city encroaching on a rural region). The relevance of specific pressures can depend on a variety of factors, such as: expected impacts (economic, ecological, etc.), connection with the key functions identified in step 1, policy or societal salience, relation with the focus of the management plans or of the study itself (e.g. evaluation of climate adaptation plans will focus on climate change; evaluation of generic plans will be much broader in scope), and potential interactions among pressures (e.g. are they mutually reinforcing?). Ideally, this characterization should be based on locally/regionally specific impact studies. More generic information, such as (inter)national scenario or impact studies can be used as well, but will need to be translated to the system-level using expert judgement (cf. De Franca Doria et al. 2009; Jonsson et al. 2011; Runge et al. 2011). Expert should be interpreted in a broad sense, including local practitioners with knowledge on the system under study.

### Step 3: Inventory of (planned or potential) management options for the region

Regional management options can be inventoried in a variety of ways, depending on the situation and the focus of the study. In many cases, some plans will already be available for the region. They may include formal regional management plans, regional visions by regional or local authorities, or business or development plans of stakeholders in the area. A relatively straightforward form of assessing resilience implications would be to test any formal plans for the region. More extensive studies could inventory stakeholder plans or wishes using document analysis and interviews, up to a full inventory of potential future developments using surveys, interviews, and workshops involving stakeholder and citizen participation.

### Step 4: Assessment of the resilience implications of the options

The implications of planned, proposed or potential management options on the resilience of the region and the key functions will need to be assessed. Approaches to assessing resilience are still in early development, and range from descriptive to quantitative. In policy practice, approaches that stay at the level of simply describing actions that are believed to benefit resilience are not unheard of. In research, more advanced and detailed, descriptive analytical approaches have been developed (e.g. Resilience Alliance 2010), as well as quantitative approaches for specific applications, such as disaster resilience indicators (e.g. Cutter et al. 2010) or flood resilience/robustness models (e.g. Mens 2015). See the Discussion (par. 5.1) and Quinlan et al. (2016) for more reflection. Whether qualitative or quantitative, it is important to distinguish between options in terms of whether they enhance or reduce resilience, and the ways in which they achieve this.

The method presented in this paper uses a set of ‘resilience principles’ to screen the implications of regional management options on resilience. These principles describe specific mechanisms by which systems can absorb disturbances and retain identity (i.e. can be resilient). Enhancing or reducing these mechanisms would correspondingly enhance or reduce system resilience. An advantage of using such principles is that they match the ways in which management options could act on the system. Assessing the implications is therefore relatively straightforward; comparing the options mechanisms of effect to the mechanisms described in the resilience principles. The resilience principles used in this paper originate in the system dynamics literature, and have been applied successfully to generate and categorize resilience-oriented climate adaptation options (Watt and Craig 1986; Wildavsky 1988; Barnett 2001; Wardekker et al. 2010):

- *Homeostasis*: multiple feedback loops counteract disturbances and stabilize the system.
- *Omnivory*: vulnerability is reduced by diversification of resources and means. It is similar to redundancy, but entails multiple different approaches that can be used alongside each other, rather than multiple copies of one approach.
- *High flux*: a fast rate of movement of resources through the system ensures fast mobilization of these resources to cope with perturbations. High flux allows for quick responses to threats and changes.
- *Flatness*: the hierarchical levels relative to the base should not be top-heavy. Overly hierarchical systems with no local formal mandate and competence to act are too inflexible and too slow to cope with surprise and to rapidly implement non-standard highly local responses.
- *Buffering*: essential capacities are over-dimensioned such that critical thresholds are less likely to be crossed.
- *Redundancy*: overlapping functions; if one fails, others can take over.

This set of principles was used because it has a solid foundation in system dynamics, describes relatively generic mechanisms, and has shown to be applicable to multiple climate-related impacts. Consequently, it can be used for a wide, ‘all (climate) hazards’ assessment. Alternative, though overlapping, sets of principles are available in the literature for various applications, such as urban planning (Eraydin and Tasan-Kok 2013) or ecosystem services (Biggs et al. 2015).

The resilience principles are used as decision criteria: each option is scored on each principle. Options were scored using a five-point ordinal scale ranging from ‘highly reducing’ to ‘highly increasing’ resilience. The collection of scores provides insight into the main mechanisms of the management plan (principles on which many options score well), as well as potential blind spots (principles on which few options provide satisfactory scores) and possible unintended negative impacts of specific options on the region’s resilience to climate change (i.e. ‘reducing’ scores). An aggregated resilience score can then be generated for each option, by taking the median^1^ of the scores on the six principles. This approach is similar to that of Gupta et al. (2010), who assess adaptive capacity using multiple principles with ordinal scales. The final resilience scores distinguish options which are particularly beneficial or detrimental to resilience.

### Step 5: Follow-up or supplementary analysis

Depending on the interests in the specific study, or the results of the assessment, it may be valuable or necessary to conduct additional assessments. These could be supplementary assessments or metrics, conducted in conjunction with the assessment of resilience implications (Step 4). One example would be to expand the set of resilience principles with other criteria into a broader Multi-Criteria Analysis. This would allow the analyst to include broader societal or policy considerations, such as costs, technical or political feasibility, time constraints, and negative and positive side-effects. Another option would be to conduct supplementary analysis after the results of an initial resilience assessment. For example, when exploring the need for options that enhance buffering, one may want to explore the required or feasible dimensions (and associated costs versus benefits) of the buffer capacity. Similarly, it could be important to explore public support for specific options if they appear to be highly beneficial for resilience, but have drawbacks. Follow-ups can be qualitative or quantitative, involving methods ranging from interviews and workshops to indicator studies or modelling.

### Case study application, data and methods

We applied the approach described above in a case study in the Venen-Vechtstreek in the Netherlands, a peat grassland wetlands system in the mid-west of the country. A case study has been developed to illustrate our approach.

Characterization of the area was performed by examining policy documents of decision makers and stakeholders in the area, as well as four “helicopter interviews” (Hajer, 1995) (provincial authorities, water authorities, and two researchers).

Characterization of the key disturbances was performed using existing national climate change scenarios and impacts studies (Van Minnen et al. 2013; KNMI 2014), as well as more detailed spatial and local impact studies (IPO 2009; KNMI 2009; Verhoeven et al. 2012; Veraart et al. 2014), and the four helicopter interviews.

Management options were inventoried in two steps. A list of currently planned options was drafted based on existing plans, particularly the regional management covenant which had already been developed by the region’s stakeholders (Stichting Ontwikkeling De Venen 2010), as well as other policy documents. This list was supplemented with options that could contribute to the area’s overall resilience, collected through literature review and interviews (the four helicopter interviews, plus three additional ones with provincial authorities, an environmental NGO, and a researcher).

The implications assessment was also performed in two steps. First, a qualitative assessment was made by the research team on the full set of options, using the information gained from the interviews and the literature. Second, a participatory assessment was conducted with stakeholders and experts. Seven people took part in the participatory assessment (provincial authorities, forestry and nature service, environmental NGO, and four scientists); three by e-mail, four during a workshop with the research team. A selection of options for agriculture and nature was assessed. The resilience principles were supplemented with additional criteria in a small-scale Multiple-Criteria Analysis (MCA) (e.g. Guitouni and Martel 1998; Huang et al. 2011), applying Multi-Attribute Utility Theory. Participants scored options on decision criteria (five-point ordinal scales), and assigned relative weights to the criteria. Criteria used were: resilience improvement (resilience principles as equally weighted subcriteria), problem urgency, no-regret characteristic, benefits, costs, feasibility, and co-benefits to other sectors.

## Illustrative case study

### Case step 1: system

#### Area description

**Fig. 2:**
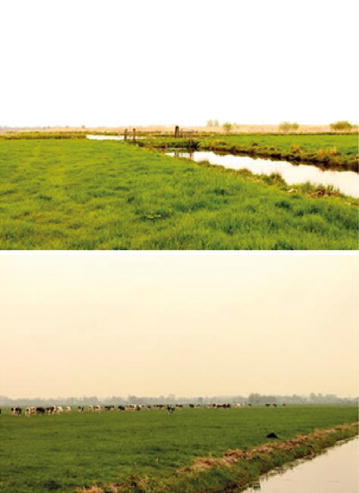
Photos of Groot Wilnis-Vinkeveen. *Top* the area is characterised by low-lying peat meadows, water, and wide, open landscapes. *Bottom* dairy farming is a key sector in the area. See Additional file 1 for more. Photos by Sara Stemberger

The case study focused on the Venen-Vechtstreek region, particularly on the Groot Wilnis-Vinkeveen area. It is a wetlands area, consisting mainly of artificially drained peat grassland and lakes, in the west of the Netherlands. The appearance of the region has changed little over the centuries and is considered ‘typically Dutch’: wide, open views, cow-filled meadows, and long and narrow stretches of land separated by water (see Fig. 2). The area is located 7 km south of Amsterdam, in the hearth of the Randstad Metropolitan Area, and is ca. 1900 ha in size. Major land use includes: agricultural grassland (76 %), freshwater (13 %), natural/wild grassland (4 %), and rural built area (3 %). See Additional file 1: S1 for photos, maps and data.

The region is part of a network of wetlands and lakes forming a ‘robust ecological corridor’ protected under Natura 2000, the European ecological network of protected nature areas. These areas were intended to be part of the National Ecological Network, although establishing the NEN proved, and will likely continue to be difficult (cf. Bakker et al. 2015). Ecological corridors have been planned to connect protected areas and strengthen the robustness of the NEN (VROM 2004, 2006). They provide connections and shelters, allowing animals and plants to migrate between nature areas.

Groot Wilnis-Vinkeveen is situated at averagely 2.5 m below sea level and has to be artificially drained to keep the land usable. Currently, the land is subsiding at up to 12 mm/year, due to peat compaction, which is intensified by the artificial draining (Stichting Ontwikkeling De Venen 2010). Different functions in the area require different levels of drainage. Wetland nature, for example, requires high water tables, while agriculture requires low water tables. This has resulted in a continual conflict between these two functions.

#### Key functions

Key functions in the area should be prioritised for resilience building. This selection should be made by local actors. In our case, they had already been clearly defined in the management covenant of the area. This management covenant was the product of collaboration and negotiation by various local stakeholders. It states as ambition that the area should be preserved as an open landscape of peat grassland in which the dairy sector can continue to develop in the future (Stichting Ontwikkeling De Venen 2010). Based on the covenant, five key functions in the area are defined:

- Clean water
- Multifaceted nature
- Providing space for a vital agricultural sector
- Providing space for a vital recreational sector

The goals pertaining to water quality focus primarily on providing sufficient clean water for nature in the study area and surrounding region. Clean water also benefits agriculture, for instance for irrigation and preventing saline seepage (Veraart et al. 2014). Water quality is impacted through import of water of lesser quality from outside sources, such as the river Rhine, as well as through local sources of pollution, such as agriculture. The current water system does have large self-cleaning capacity, primarily due to its large surface area.

Multifaceted wetland nature is an important function. The area contains valuable water, riparian and arid land plant species, is a haven for otters and numerous species of meadow birds. The combination of water and land offers chances for nature, and the water system has a large self-cleaning capacity. Due to differences in artificial drainage, the nature areas are now higher than agricultural areas. Therefore, nutrient poor water from nature areas now flows away to lower areas, and has to be replaced with water from outside the region. This makes the natural areas vulnerable to the, often lesser, water quality of these sources (Veraart et al. 2014).

Agriculture, particularly dairy farming, is the most important economic factor in the region and should remain economically viable. It is also highly important for the heritage value of the peat grassland landscape. The availability of suitable land is a key issue, particularly in relation to soil subsidence and attempts to reduce this through reducing the level of drainage. Agriculture requires substantial drainage. Reasonably dry soil is important for access to the land (e.g. heavy machines), as well as for the cows and grass. Agriculture also effects soil quality, water quality and biodiversity.

The recreational sector should focus on tourism utilizing the wide, expansive views and befitting the nature and functions of the peat grassland landscape, particularly agriculture. Recreational development should follow the goals for nature, agriculture, and water. The area can offer peace and quiet and space for visitors from the surrounding urban areas, with an emphasis on authenticity and heritage. Tourism may in turn lead to more support for preserving the region. Rural tourism and activities such as walking, cycling, and canoeing are particularly important.

### Case Step 2: Key disturbances due to climate change

Climate change is one of the key disturbances in the area for the coming decades. This section will briefly discuss impacts on the key functions. More details can be found in Additional file 1: S2.

Climate change can reduce water quality and lead to changes in water availability: decrease or increase in summer, increase in winter and increase in heavy precipitation (KNMI 2014). Increased water temperatures can strongly degrade the ecological quality of the surface waters. Heat, drought, and heavy precipitation can lead to more nutrients being released into surface water. Eutrophication and heat can in turn increase microbial contaminations and algae blooms (IPO 2009; Verhoeven et al. 2012). Drought, hence reduction of water volume, can also build-up pollutant concentrations.

Nature is affected through water temperature, availability, and quality (Verhoeven et al. 2012): eutrophication poses a moderate risk to aquatic nature, but small risk to terrestrial nature; drought risk is moderate to high. Changing climatic conditions will also influence the distribution of species (e.g. IPCC 2014), forcing species to shift to other areas. Changes in growing seasons may also change the timing of peak distributions of plants, insects, and animals. This can lead to mismatches in food availability (Van Minnen et al. 2013). The ecological impacts of this are not yet clear.

Agriculture is affected through changes in precipitation, evaporation, and drought conditions. For the study area, this can increase the potential precipitation deficit during summer (KNMI 2009). Prolonged warmth and droughts reduce water availability and reduce grass quality. Heat and drought negatively impact dairy production. Intense or prolonged rainfall reduces the accessibility and usability of agricultural lands. The longer growing season and increased CO_2_ concentrations may have some beneficial effects due to higher grass yield.

Recreation may be affected by the impacts described above as well (cf. Van Minnen et al. 2013). Drought, heat, low water quality and levels, and potential negative effects on the landscape (via impacts on agriculture and nature) could be negative for recreation. Positive effects can also be expected, as the number of favourable days for outdoor recreation will increase, due to warmer weather, and residents from the surrounding Randstad cities may seek refreshment in the area during warm days.

### Case Step 3: Management options for the area

Potential management options were inventoried, based on the case-study area’s management covenant (Stichting Ontwikkeling De Venen 2010). The options that were proposed in the covenant were divided in options taken to improve agriculture, nature, recreation and clean water (see Table 1). However, note that options can have cross-category consequences. For instance, some nature-oriented options would have serious implications for agricultural entrepreneurs. The list of options from the covenant was geared towards those options that improve the health of the key functions in general, which might not cover all options that relate to improving climate resilience. Therefore, a secondary inventory was performed, focused on options for improving climate resilience specifically, based on the initial helicopter and later in-depth interviews, and the expertise available in the research team.

**Table 1:** Planned management options for Groot Wilnis-Vinkeveen (Stichting Ontwikkeling De Venen 2010) and their potential implications

Option in management covenant	Option in case study	Description and potential implications
Agriculture		
Subsidising plot dams/culverts	A1	Option for optimising the plot arrangement and accessibility. It would allow for faster movement over the agricultural land in times of flooding, increasing high flux. It also increases the options for water management in the region: there are more ways to get water in or out, improving redundancy
New, additional pumping station at Korenmolenweg	(included in A1)	Option for optimising plot accessibility. It can be part of a feedback system to keep the water level constant, improving homeostasis
Subsidising (pilots for) under water drainage	A2	Option for optimising plot accessibility. It increases the ability of agriculture to handle higher water tables in a dynamic way (homeostasis), and limits soil subsidence. It also spreads the water dependence of the drained plot over multiple water sources, increasing omnivory. Potential co-benefit is that limiting soil subsidence is also good for natural areas. Potential negative side effects could take place on meadow birds and local water quality
Realisation of balanced fertilization, earlier than currently obligatory	A3	Using only the amount and type of fertilizer that is necessary, will lead to less eutrophication and possibly lower costs for the farmer. Co-benefits: cleaner water, potentially higher biodiversity on agricultural land and in the water
Stimulating the switch to biological farming	A4	Biological farming uses ecosystem feedbacks to control pests (homeostasis). It could also help dairy farmers improve their chances of survival in the future, e.g. by being more responsive to current societal trends (homeostasis), and less dependent on marginal markets (buffering). If applied in a broader way (beyond only fertilizers and pesticides) to stimulate diversification of production, it could increase omnivory. Could present many co-benefits: for recreation, clean water, biodiversity, health of humans and animals, and reduced CO_2_ footprint
Stimulating business upscaling	–	Option to increase economic viability of farms. It would result in larger, but fewer companies, which would reduce the redundancy of the region: if a company runs into problems, a larger fraction of the economy would be impacted. The measure may make it easier for large companies to mobilise resources for innovations and adaptations, increasing high flux. It could entail co-benefits for water quality, if it also increases the availability of resources for environmental management on farms (e.g. limiting nutrient leaching)
Employing an area broker for agrarian entrepreneurs	–	Option for stimulating and supporting entrepreneurship. Provides knowledge and connections that can stimulate agrarians to offer ‘green and blue services’ (omnivory), and improve communication among actors (high flux). It may have co-benefits for recreation, nature, and water quality
Subsidising business plans aimed at making business more sustainable and diverse (e.g. green and blue services and agro-tourism)	–	Option for stimulating and supporting entrepreneurship. Increases omnivory and flatness, because it will make agribusinesses more diverse, with a broader income base. It could also improve homeostasis, assuming it will increase the synergy between green services, blue services, dairy farming and tourism. Co-benefits: good for tourism and nature
Nature		
In the core area for meadow birds, a marshland connection will be realised through a diffuse system of capillaries passing existing waterways, with natural banks of at least 5 m wide and containing several, spread out ‘marshland beads’	N1	The marshland connection and capillaries will provide a habitat for many species and (alternative) corridors through which other species can migrate (redundancy, homeostasis), including animals and plants (Soomers et al. 2013). It also increases water buffering. It will have co-benefits: cleaner water (marshland filters the water), and enhanced recreation
New (marshland) nature will be realised on the lowest plots. About 50 ha, 20 % of water level Sect. 9	(included in N1)	Water level Sect. 9 is the lowest area in elevation; i.e. hardest to keep dry for agricultural purposes, and easiest to turn into marshland nature. Conversion to marshland will reduce the need for pumping (lower quality) water from low-lying agricultural lands into higher nature areas, stimulating and reducing pressure on natural processes (homeostasis). The area can also serve as water buffer. This has co-benefits for agriculture, water management and quality, and reduce subsidence. Moving farmers will require much effort (e.g., cooperation, work, finances)
For the combined marsh- and wetland connection, a single width (100 m) of marshland or wetland chain will be constructed, instead of the sum of two widths	(included in N1)	Marshland and wetland are good for water quality, because they improve natural water cleaning (homeostasis). They also improve water retention (buffering), which limits drought and flood related problems. These effects have co-benefits for nature and agriculture. The options also adds variation to the landscape, which has co-benefits for recreation
Dredging all waterways, increasing water depth to 0.5 m in secondary and tertiary waterways	N2	Can be beneficial for aquatic macroinvertebrates, but is in conflict with having natural banks. Perhaps a minor recreation benefit: better access by canoe
Stimulating natural dynamics in water levels, in part by retaining water on shallow, partly dug-off, natural banks. Where this conflicts with agrarian use, alternative measures will be taken to achieve comparable conditions	N3	Certain valuable bank plants require reasonably natural dynamics in water level. This can be enhanced by retaining rain water on shallow, partly dug off, natural banks (relates to homeostasis). Natural banks can also filter water (homeostasis) and improve water buffering. They have co-benefits for clean water and recreation
The inlet of water is reduced to a minimum	N5	This reduces the intake of water with lower water quality from neighbouring areas, therefore reducing pressure on the natural water cleaning capacity (removing existing negative impacts on homeostasis). This benefits water quality and nature. However, it also involves a risk of keeping contaminants in the system for a longer period of time (reducing high flux)
The nature goals are fitted optimally into the existing landscape. Because swamp and brushwood (part of the wetland goal) don’t fit in the open peat-meadow landscape and clash with meadow birds and farming, the robust nature connection is split up into two different routes	–	Two different routes, with different characteristics, provide ways for species to migrate and move across the region. As some species may be able to migrate using either route, this could increase omnivory. It may however make individual routes more vulnerable, because they are only half as wide. Some recreation co-benefits: it keeps nature development in line with current open landscape, which is a key characteristic and main selling point of the region
Water Quality		
Realisation of natural banks (35 km)	(included in N3)	Vegetation cleans the water by filtering (homeostasis). It involves co-benefits for nature as it provides a habitat for species, and co-benefits for recreation as it increases the area’s aesthetic value
An extra main water-course	N4	This would allow water to be moved faster, either out of the area in case of flooding, or to the area in case of drought (high flux). The water-course itself can be a buffer, if water can be stored in it for a while (buffering)
Recreation		
Canoe route: through Donkereind and Demmerik and de Geuzensloot	–	This option could increase homeostasis: visits and income from tourists will depend on the attractiveness of the area, providing an impetus for policymakers to address climate change impacts and other pressures. It does put pressure on nature and water quality, but this is limited if it only involves quiet, ‘muscle-powered’ recreation (no speedboats, etc.). Primary effect may be as co-benefit to other functions, e.g. nature (natural beauty is important for visitors), water quality (as previous), and agriculture (diversifying sources of income)
Hiking and biking routes: through the nature reserves	–	As previous

### Case Step 4: Resilience implications

A first, qualitative (descriptive) analysis of the list of the potential impacts of the options suggested in the management covenant (Table 1) was performed for all four key functions. The research team did this analysis, using the set of resilience principles. This provided some initial indications of how these options would influence resilience and potential priorities for a more in-depth participatory assessment. We also noticed that many options have side-effects, such as influence on the resilience of key functions other than the ones they were assessed on, as well as co-benefits and feasibility-related concerns. This provided input for a supplementary multi-criteria assessment to be added to the participatory resilience assessment, in Step 5 (par. 3.5). The results of the qualitative analysis can be found in Additional file 1: S3).

Following the qualitative analysis, we invited a group of practitioners and experts who had experience in the area, as well as several resilience experts, to perform a semi-quantitative (ordinal scale) analysis in a workshop. Based on the qualitative data, this analysis was limited to options for nature and agriculture. It included options from the management covenant as well as the supplementary set developed in Step 3 (par. 3.3) (suggested with resilience in mind).

The workshop took the following form. The participants introduced themselves before the workshop and indicated their background, expertise, and interests. Each of them contributed with knowledge and opinions, and some provided additional information (e.g. maps). First, a general introduction to resilience, the case study area, and the topic of climate resilience in the case study area was provided. The results of the research already conducted (climate change impacts inventory, options inventory) were also presented. Second, we discussed the notion of resilience principles with the participants and jointly reflected on what these could mean for the case study area, and the key functions of nature and agriculture specifically. The concept was easily brought to their attention, although part of this ease could be explained by the fact that they all had experience in environmental management (exploring the concept of resilience with citizens or farmers, for instance, would be more difficult and would require more effort in both preparation and execution of the workshop). This step helped bring resilience and resilience principles from relatively conceptual notions and mechanisms to a more practical understanding that the participants felt comfortable to work with. The combination of practitioners, local scientists, and resilience scholars was particularly helpful in discussing and translating the principles from conceptual to practical notions (see Table 2).

**Table 2:** Translation of resilience principles to potential practical interpretations for agriculture and nature

Resilience principle	Mechanism	Primary mechanism (s)	Potential Practical interpretation (for key functions in the case study)
Buffering	Essential capacities are over-dimensioned such that critical thresholds are less likely to be crossed	Absorbing disturbances	*Agriculture*: Agrarians have sufficient resources to be able to handle disruptions to the ‘normal’ situation and longer difficult periods (e.g. prolonged heat/drought)
			*Nature*: There is sufficient capacity (e.g. area of land, number of individuals or species, refuges, etc.) and ecological memory to be able to cope with climatic shocks and long term stresses, so that typical types of nature can recover after disturbances and/or do not get eroded beyond repair
Redundancy	Overlapping functions; if one fails, others can take over. Multiple copies of one approach, function, or service	Absorbing disturbances	*Agriculture*: Agrarians have backup options to maintain running farms and a functioning agricultural sector in the area during disruptions. If one specific aspect of a farm or the area is affected, it does not disrupt the entire system
			*Nature*: Nature areas are compartmentalised and populations are sufficiently large to be able to handle situations where specific locations or individuals fall away (e.g. are destroyed or killed) due to a disturbance
Omnivory	Diversification of resources and means. Multiple different approaches that can be used alongside each other, rather than copies of one approach	Absorbing disturbances, recovery, reorganization	*Agriculture*: There are multiple ways to maintain the agricultural function in the area and agrarians have a variety of ways to cope with shocks and stresses (e.g. diversity of resources or means of income). If one falls away, other options are available
			*Nature*: There is sufficient biodiversity available in the area to maintain the desirable nature types in the area. Multiple types of species fulfil the same ecological function, or there are diverse ways to cope with any shocks or changes
Flatness	The hierarchical levels relative to the base should not be top-heavy. Systems without no local competence and mandate to act are too inflexible and slow to cope with surprise	Quick response, self-organisation	*Agriculture*: There is close interaction between agrarians and authorities and other decision-makers relevant to the region, and/or agrarians have the means and authority to act in response to disturbances themselves
			*Nature*: Nature managers and other people involved in the practical maintenance and functioning of the natural areas should be as direct as possible (e.g. no complex lines of responsibility, long decision chains), so early warning signs and suggestions for improvement are picked up rapidly. Food pyramids in the area should not be taller than the area allows
Homeostasis	Multiple feedback loops counteract disturbances and stabilize the system	Quick response, self-organisation, learning	*Agriculture*: Mechanisms are in place to monitor the functioning and health of regional agriculture, spot (potential) problems, and quickly and creatively act upon these. Including economic health, as well as long term matters such as embeddedness in the area’s other functions
			*Nature*: There are clear lines of interaction among nature managers and with other relevant parties (e.g. farmers, recreation sector, water sector). Procedures and mechanisms are in place to monitor the area’s health, spot problems, and act upon these. Natural dynamics through which local nature copes with shocks and stresses are stimulated and maintained
High flux	Fast rate of movement of resources through the system ensures quick mobilization to respond to threats and changes	Quick response, recovery, reorganization	*Agriculture*: Resources (e.g. money, land, buildings, knowledge, ideas) can be quickly mobilised to cope with shocks and stresses and adapt to changes. They are not tied up too much in capital. Adaptive capacity is high
			*Nature*: Resources (e.g. money, nutrients, biological components, water, knowledge, ideas) can be quickly mobilised to cope with shocks and stresses and adapt to changes. Natural dynamics and processes are rapid and unhindered; adaptive capacity is high

Third, participants made a selection of key options that they thought were most important or most interesting to prioritise in the analysis, given the limited available time. They merged several of the options, as they felt they overlapped (noted in Table 1). Participants were provided with score forms/matrices (one per option) that included the six resilience principles, as well as a preliminary set of other criteria (for the MCA in Step 5), with a standard five-point Likert scale to rate the impacts on the resilience of their associated key function. Each option was discussed plenary, then scored individually. As noted in paragraph 2.6, several stakeholders who were unable to attend the workshop were visited afterwards to conduct in-depth interviews and retrieve their input. While they could not participate in the discussions, an advantage was that the operationalisation and further defining and refining of the principles, criteria and options had already taken place and the participants could work with these right away. We also used these interviews to reflect on the methodology and scoring of options in the workshop, which provided useful material to develop this paper.

**Fig. 3:**
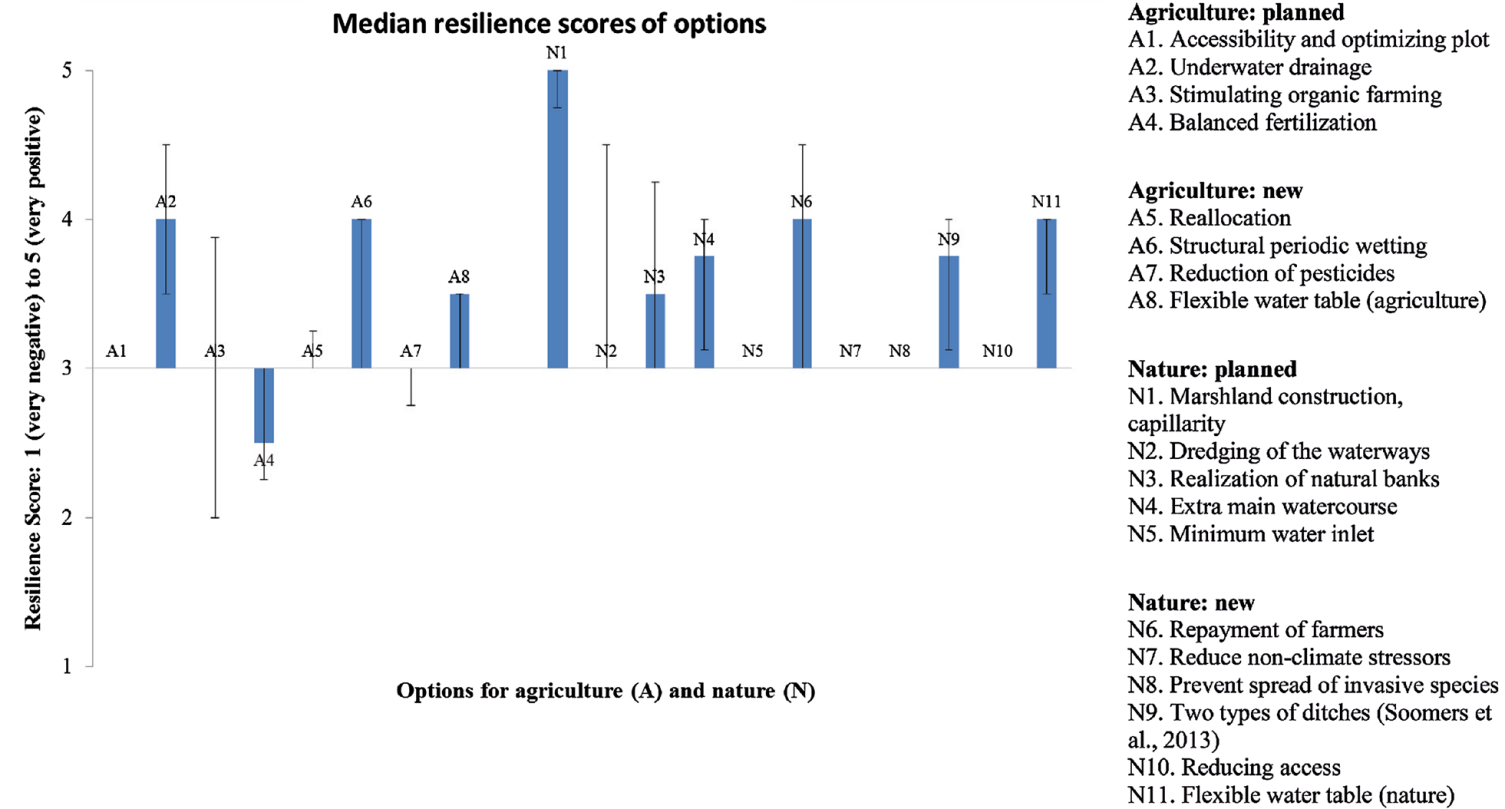
Median resilience scores of options for agriculture and nature. The *error bar* indicates the interquartile range of individual median scores

For nature, six out of eleven had positive (score >3) median impacts on resilience; for agriculture three out of eight (Fig. 3). Many options had a neutral (score 3) median impact. The participants expected the option ‘marshland construction and capillarity’ (N1) to perform particularly well. Other well-scoring options were: underwater drainage (A1), structural periodic wetting (A6), repayment of farmers for ecological services (N6), and flexible water table (nature) (N11). The newly suggested options (suggested with resilience in mind) performed only marginally better, on average, than those already planned (suggested to improve the health of the key function in general): see the scores in Fig. 3.

**Fig. 4:**
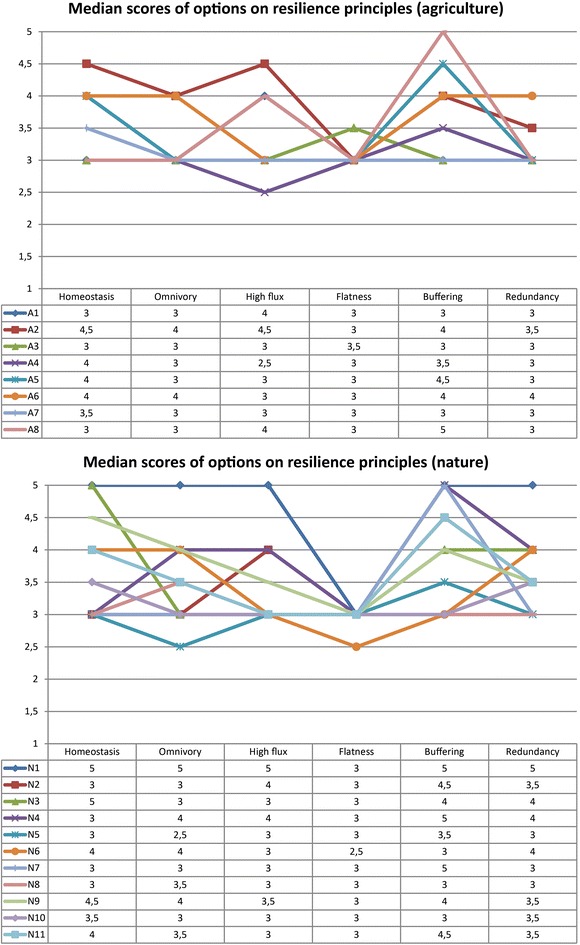
Resilience scores of options, disaggregated per resilience principle

Both the marginal difference between non-resilience and resilience-based options and the large number of options that had neutral impact on resilience (in both categories) were surprising. However, one should be aware that these are median scores, averaged over the participants and the six principles. Often, such overarching metrics can average out more insightful variations, hidden in the disaggregated scores (cf. Wilk and Jonsson 2013). The scores on separate resilience principles can provide a good way to diagnose specifically how different options influence resilience. On separate resilience principles, all options had a positive median score on at least one principle (Fig. 4). Several options had a small median negative impacts (2.5) on single principles: balanced fertilization (A4) on flux, minimize water inlet (N5) on omnivory, and repayment of farmers (N6) on flatness. Most notably, the flatness principle remained unutilised by nearly all options. Only one option, organic farming (A3), had positive impact on flatness. Several other principles were also underrepresented by the options: redundancy and omnivory for agriculture (each 2 of 8 options), and high flux for nature (4 of 11 options).

Balanced fertilization (A4) received a negative median score, and for stimulating organic farming (A3) the interquartile range was partly negative. It should be noted that, while these are agricultural options, their main goal in the covenant is to improve nature. For both, individual scores ranged widely (1–5). Neither the scores on separate principles (no clear pattern) nor the argumentation (very little given) provided much clarification. Several participants giving positive scores indicated that fertilization would become more attuned to local needs (homeostasis), and that introducing organic farming would lead to more diversity in the sector (omnivory). A ‘moderate’ participant suggested that A4 would be expensive precision agriculture, and that A3 might be detrimental to agriculture in the short term, but beneficial in the long run.

The interquartile ranges for total resilience and individual principles were 1–2 points. The group size was sufficient for medians and interquartiles to be robust even to single participants scoring notably different. Exceptions occurred, in the above cases, when multiple participants strongly diverged in opinions. Dredging of waterways (N2) and repayment of farmers (N6) also sort fairly wide interquartiles. N6 due to ranging individual scores and N2 caused by differences in the number of principles receiving positive scores: buffering and high flux received high scores from most people, while opinions diverged on others.

### Case Step 5: Follow-up using multi-criteria analysis

**Fig. 5:**
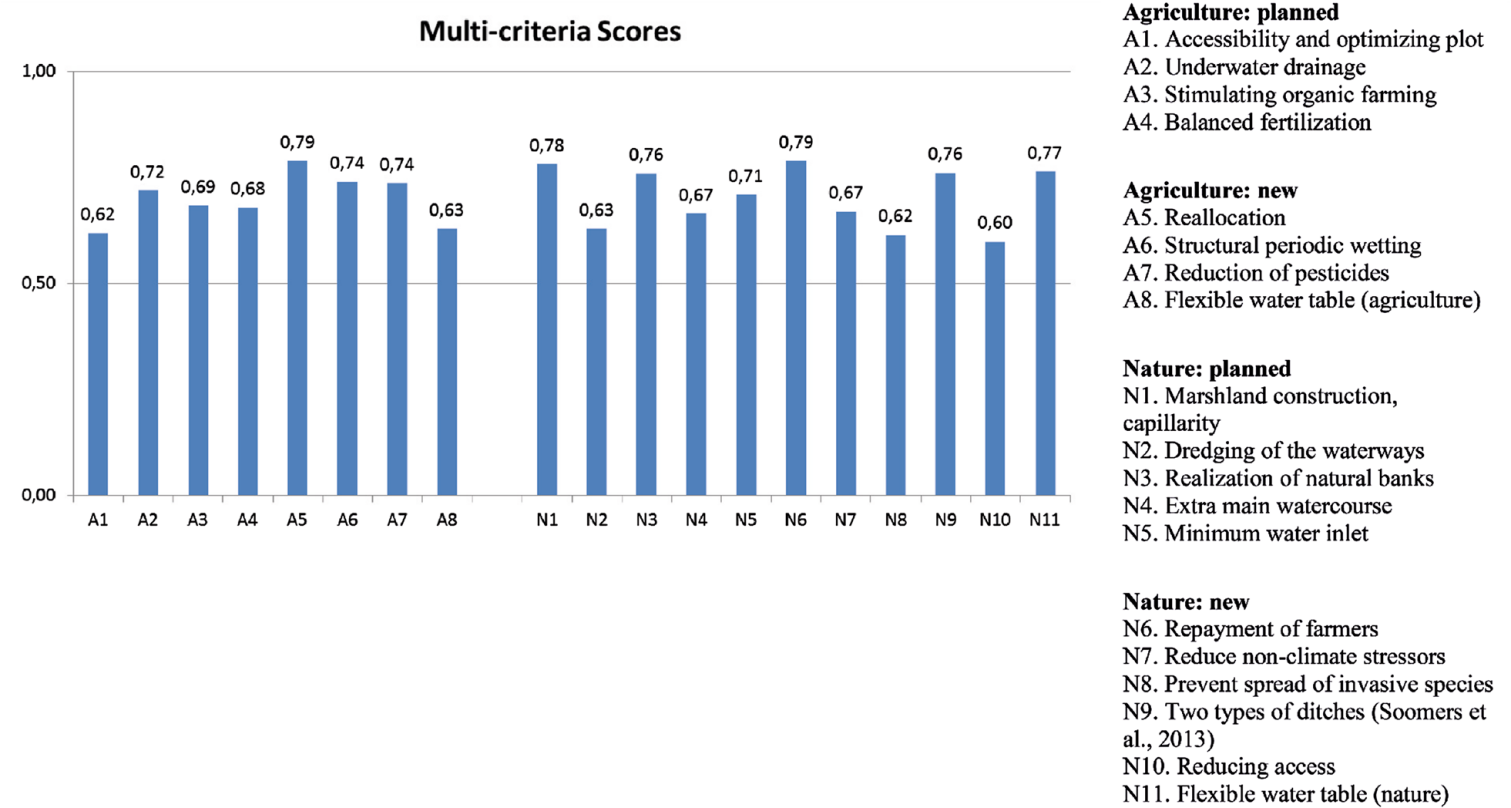
Multi-criteria analysis, using seven criteria

For illustration, we performed a small MCA (Fig. 5), in which the resilience principles were supplemented with other criteria. Several criteria were suggested by the research team. Participants discussed, supplemented and selected the final set. The score form/matrix explicitly left room for this. Participants selected the following criteria, and assigned the following relative weights to these criteria: resilience improvement (30 %), co-benefits (20 %), urgency (20 %), benefits (7.5 %), costs (7.5 %), no-regret (7.5 %), and feasibility (7.5 %). Resilience improvement received the highest priority, as it was the main goal of this study. This criterion consisted of the six resilience principles, as subcriteria with equal weight. Co-benefits was judged second-most important, due to the successful policy in the area, strongly dependent on the decision-maker’s ability to convince farmers and other stakeholders of the options usefulness. The more co-benefits, the less resistance to options not directly in their benefit.

For nature, marshland construction (N1) scored high (0.78; 0–1 scale), as did repayment of farmers for nature services (N6; 0.79) and flexible water table (N11; 0.77). For agriculture, reallocation of agriculture and nature (A5; 0.79), structural periodic wetting (A6; 0.64), and reducing pesticides (A7; 0.74) scored well. Compared to the resilience assessment, repayment and reallocation now roughly equal marshland construction, due to high scores on co-benefits and urgency.

## Discussion

### Comparison with other approaches

Our approach to assessing resilience implications is semi-quantitative; an ordinal scale valuation on resilience principles. Examples of quantitative approaches are available as well. For instance, Cutter et al. (2010) explore disaster resilience with a large set of indicators, and Mens (2015) models flood and drought robustness (resistance plus resilience). Thissen et al. (2015) compare ordinal analysis, robustness modelling, and exploratory modelling for a freshwater system. They conclude that robustness modelling requires considerable computation and quantitative input and understanding of the system. Exploratory modelling also requires significant computation, but less probabilistic specification of input. A qualitative or semi-quantitative resilience approach requires little quantitative input, can easily include stakeholder perspectives and tailoring to local situations, but can’t assess the efficiency of options and is less spatially explicit. Thissen et al. conclude that ordinal/qualitative approaches are useful for large-scale applications with multiple impacts, sectors, and stakeholder processes. Quantitative approaches are suitable for more narrowly constrained situations, such as the vulnerability of a specific sector to a specific impact. Indicator-based studies could provide a middle way, trading the speed of our approach for better quantitative and (potentially) spatially explicit evaluation. They do depend on the availability of suitable indicators and data to evaluate them. It would also be easier to develop a baseline of resilience than to evaluate the impacts of newly proposed policy options. Quantitative approaches are resource-intensive. A screening using our approach could provide an indication regarding what options or functions require a quantitative follow-up. Other tools also could be considered to further evaluate options, such as Multi-Criteria Analysis, cost-benefit analysis, or deliberative tools. Their suitability depends on the case-specifics, including framing of the adaptation problem, uncertainties and decision-strategy (Wardekker et al. 2009; De Boer et al. 2010). In our study, MCA usefully indicated that co-benefits was an important additional criterion for societal support.

Our approach bears some similarities with the concept of ecosystem services (e.g. Carpenter et al. 2009; Millennium Ecosystem Assessment 2005; Bouma and Van Beukering 2015). It relates to the notion that ecosystems provide value to society by providing various services and resources, such as water storage and purification, fertile soils, biomass, and recreational opportunities. Consequently, ecosystems are valuable and worth protecting—payments could be asked for such services, which could in turn be used to manage the ecosystem. This notion is very similar to our use of ‘key functions’ of a system. Those in the case study could be interpreted in this way. An important difference is that our approach converge on what actors value in an area or system as a whole—not only the ecosystem. We also do not apply (economic) valuation of functions, but such methods could be useful to explore priorities and trade-offs between functions.

As noted in Step 5 (Follow-up) in our approach, it is useful to consider follow-up or supplementary approaches to further refine the assessment. Many of the approaches discussed above have their own range of applicability and pros and cons. For instance, they may be best applied at different scales, have different requirements regarding data availability, and have a different scope in the types of research questions that can be answered. Similarly, both qualitative (resilience assessment) and quantitative (resilience measurement or modelling) approaches have specific value and applicability, and can often be used in a complementary way (Quinlan et al. 2016). The assessment team will need to make a careful judgement on these matters on a case-by-case basis.

### Reflection on the approach and case study results

The approach to assessing the implications of regional management options for (climate) resilience provided a relatively quick way to scan a variety of options, on a range of impacts. It also allowed us to take into account a range of stakeholders and their viewpoints.

The concept of ‘key functions’ of a system allowed us to make a distinction between the preferred system state and other potential states, that was straightforward to identify and easy for stakeholders to use. It also takes into account the notion that resilience is not by definition a ‘good’ property; it is not normative. Rather, climate adaptation would focus on enhancing the resilience of those aspects that should be maintained. This is inherently subjective and selecting ‘key functions’ should preferably be based on a participatory process. In the case study, that consensus process had taken place in the region; a management covenant with clear goals had already been established. A potential downside of the approach is that it does not provide a full account of system dynamics and components. Rather, an initial scan is made in the process of characterising the system, its history and its stakeholders. A related topic to keep in mind, is that options may enhance resilience for one function, but reduce it for another. Negative impacts on other functions are not systematically addressed. It could be accounted for by adding an additional criterion in an MCA, or by explicitly discussing this issue with participants. In the case study we accounted for it by explicitly assessing an option for which we expected mixed effects (flexible water table; A8/N11) under two functions.

The resilience principles facilitated a quick assessment, in a participatory setting. Having experts score and discuss options on the basis of such principles provides insight into possible feedback loops, overlap with other policy issues, and specific local sensitivities. In participatory approaches such as this, it is important to make sure that relevant perspectives are included. In our case study, for instance, the agricultural association was unable to participate. However, the other participants’ expertise and viewpoints were broad enough to include (or at least reflect on) their perspectives. The options and principles used will need to be clear, and a workshop setting is useful to clarify any (remaining) ambiguity, controversial details, or potential differences in impact due to different ways of implementing an option. Particularly, it is useful to have a combination of practitioners, local scientists, and resilience experts in one session, as this helps move resilience from a relatively conceptual matter to a more specific, local notion of the way the area might function.

In the case study, we evaluated options both qualitatively (describing effect on specific principles) and semi-quantitatively (ordinal 1–5 scale rating). In the ordinal analysis, both the scores on separate resilience principles and the aggregated score (median of separate principles) provided relevant information. Unaggregated scores showed that some principles were underutilised in the set of options: redundancy and omnivory for agriculture, high flux for nature, and flatness for both. It would be useful to enhance the regional plans with these principles in mind. Aggregated scores revealed that the extra options suggested with resilience in mind, did not score better than those already planned. Often, neutral-scoring new options scored well on one or two principles and neutral on others, leading to neutral median scores. This suggests that they improve resilience, but are best taken in concert with options enhancing other principles. Conversely, the more generic planned options scored well on more than two resilience principles, leading to positive median scores. This raises the question of how to interpret such scores. Aggregating by median leads to positive scores only if an option improves several resilience principles. It separates options with a broad positive impact, from those with broad negative impact, but ignores options (positively/negatively) impacting only one or two principles. Aggregation using the mean would have included this (but is mathematically incorrect for ordinal data). It also raises the question of whether a negative impact on one principle may be compensated by a positive impact on another. Alternative or complementary aggregation principles could be considered, such as discarding principles with any negative scores (satisficing), giving more weight to negative scores, giving more weight to scores of “very good/bad” versus “good/bad”, et cetera.

As a general lesson for applying this type of resilience assessment, one should note that the overall resilience score (medians, aggregated over the participants and the six resilience principles) averages out some of the more interesting differentiations that can be spotted among the disaggregated scores (cf. Wilk and Jonsson 2013). The overall resilience scores are good at distinguishing options that have a broad positive impact on resilience (i.e. positive on multiple principles) from those that have a broad negative impact. Those that are more tailored, and have a more narrow impact (i.e. influence only one or two principles) show up as ‘neutral’, which may not do justice to their actual impact. The scores on individual resilience principles provide a better diagnostic of the mechanisms that the options use to impact resilience. Since specific resilience principles influence resilience in different ways (described as ‘Primary Mechanisms’ in Table 2), this can provide important insights into any gaps or weaknesses in the package of options. For instance, some principles focus on absorbing impacts, while others enhance recovery, self-organisation, and (autonomous) adaptation. It can be telling (and possibly undesirable—but this is an interpretation that is up to the local decision-makers) if specific principles or even specific mechanisms are uncovered.

Further in depth studies could be undertaken to flesh out the details for specific issues. This could be relevant, for instance, to system components that might already be close to critical thresholds (e.g. endangered ecosystems),or management options that require more fine-grained balancing and dimensioning (e.g. determining required buffer capacities for freshwater storage).

## Conclusions

We developed and tested an approach to assess the impacts of regional management options on climate resilience. The proposed practice includes five steps: (1) characterizing the system (defining key functions), (2) characterizing the impacts of climate change and other disturbances on these key functions, (3) inventorying potential management options, (4) assessing the impacts of these on the climate resilience of the key functions, and (5) performing supplementary or follow-up analysis as the situation requires. For the resilience assessment, we used a set of six ‘resilience principles’, scored on a five-point ordinal scale (highly increasing-highly reducing). This approach provides a relatively quick way to evaluate a set of options. It allowed us to consider multiple impacts and sectors, multiple dimensions of resilience, and stakeholder perspectives. It was also easy to perform in a participatory manner, and to integrate into a multi-criteria assessment, allowing for a broader evaluation. The approach is especially useful for a prompt, broad screening of options. A scan on median impacts (averaged over the six principles) can be used to distinguish options that have a broad positive versus a broad negative impact on resilience. Scores in individual resilience principles provide a good way to diagnose exactly how (that is, by which mechanisms) specific options influence resilience. The results can then be used to identify gaps or pitfalls in the set of options considered, to prioritize options and policy packages for a more in-depth analysis. An additional advantage is that the approach educates participants in the concept of resilience and provides them with a tool that enables and stimulates resilience-thinking amongst them.

A case study was performed in the Dutch wetlands. Key functions were: agriculture, nature, clean water, and recreation. The analysis revealed that some principles were underutilized: particularly flatness, but also redundancy and omnivory for agriculture, and high flux for nature. We endorse the addition and establishment of options that score high on these principles to the management plans. Co-benefits turned out to be an important criterion to obtain support for resilience-based climate adaptation measures from local stakeholders, such as farmers.

## Additional file


10.1186/s40064-016-2408-x Supplementary material for screening regional management options for their impact on climate resilience.

## Footnotes

1 Alternative aggregation methods can be considered; see “Discussion” section.
