# Supplementary material for: Screening regional management options for their impact on climate resilience: an approach and case study in the Venen-Vechtstreek wetlands in the Netherlands
Source: Springerplus. 2016 Jun 17;5(1):750. doi: 10.1186/s40064-016-2408-x (PMC4912504; doi:10.1186/s40064-016-2408-x)
Supplement: Supplementary file 1 — 10.1186/s40064-016-2408-x Supplementary material for screening regional management options for their impact on climate resilience. [file 40064_2016_2408_MOESM1_ESM.pdf]

Supplementary Material for "Screening regional management options for their impact on climate resilience"

J.A. Wardekker, D. Wildschut, S. Stemberger, J.P. van der Sluijs, 2016.

S1. Case study area impression, maps and key data

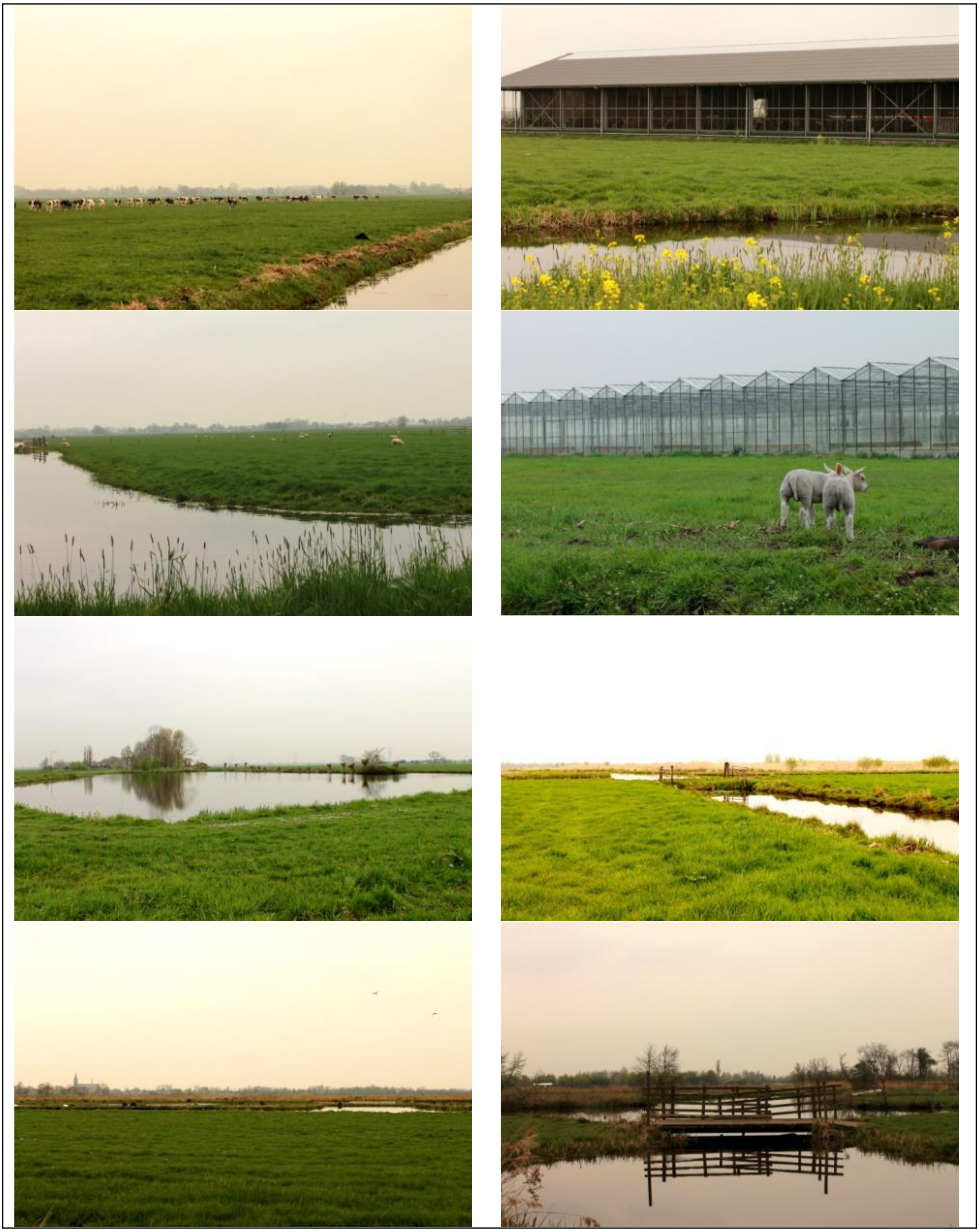

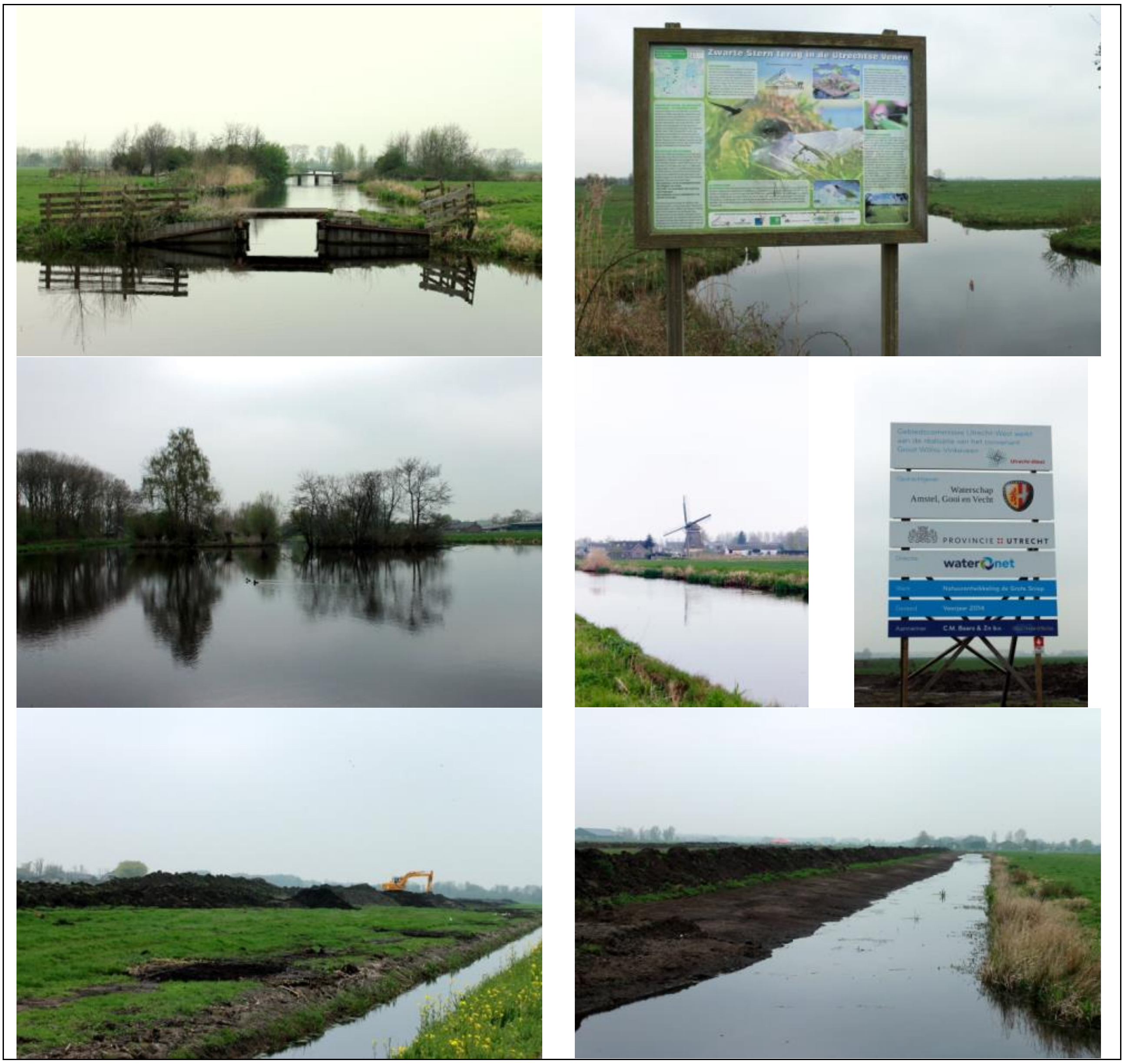

Figure S1. Photo impression of the case study area (photos by Sara Stemberger).

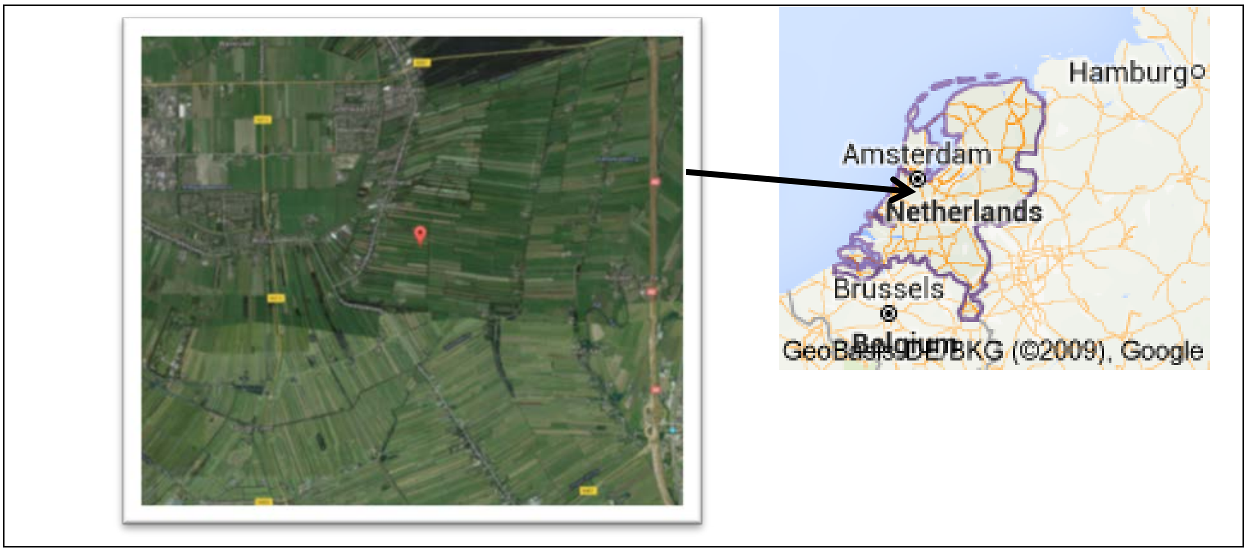

Figure S2. Satellite view and relative location of the Groot Wilnis-Vinkeveen case study area (source: Google Maps).

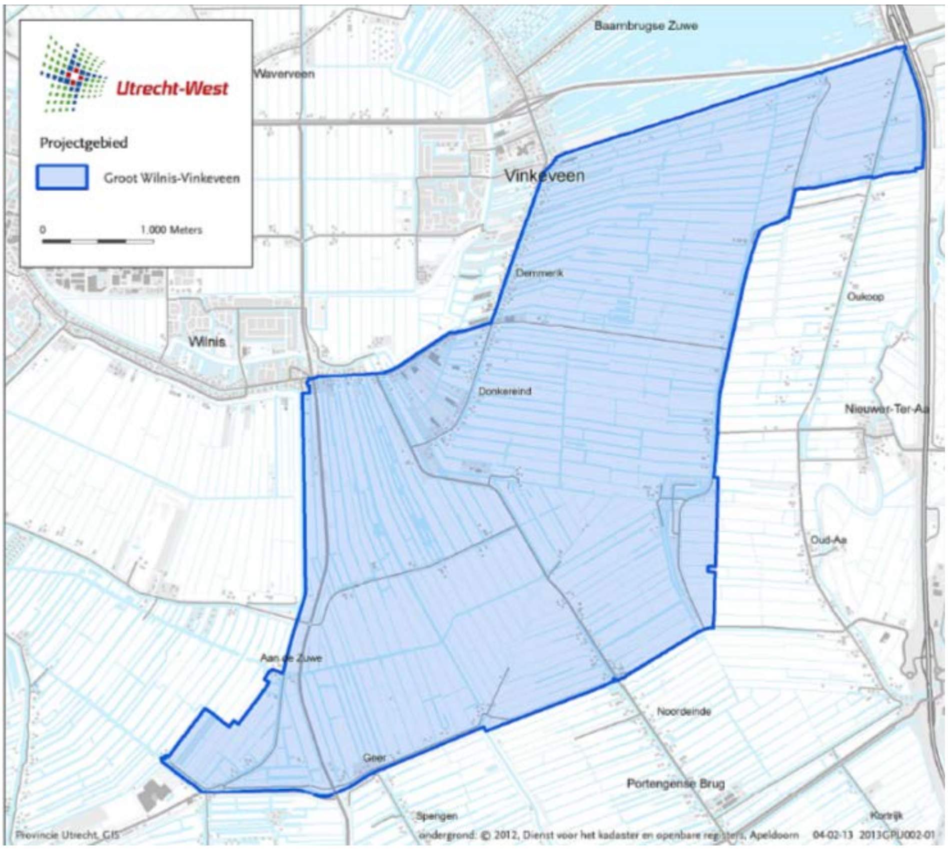

Figure S3. Boundaries of the case study area (Utrecht-West, 2012a).

Table S1. Land use in the Groot Wilnis-Vinkeveen area (estimated with ArcGIS, data of 2012; Hazeu et al., 2014).

Land Use Category	Area (ha)	Map color
01. Agrarian grassland	1450	
06. Other crops	1	
08. Greenhouses	2	
09. Orchards	1	
10. Flower bulbs	2	
11. Deciduous forest	16	
12. Coniferous forest	2	
16. Freshwater	241	
18. Buildings, primary built area	>1	
19. Buildings, secondary built area	>1	
22. Forest in secondary built area	7	
23. Grass in primary built area	1	
25. Main roads and railway	17	
26. Buildings, countryside	64	
28. Grass in secondary built area	9	
41. Other swamp vegetation	>1	
42. Reed vegetation	3	
45. Natural grasslands	83	
61. Tree nurseries	11	
62. Fruit farms	>1	
TOTAL	1909	

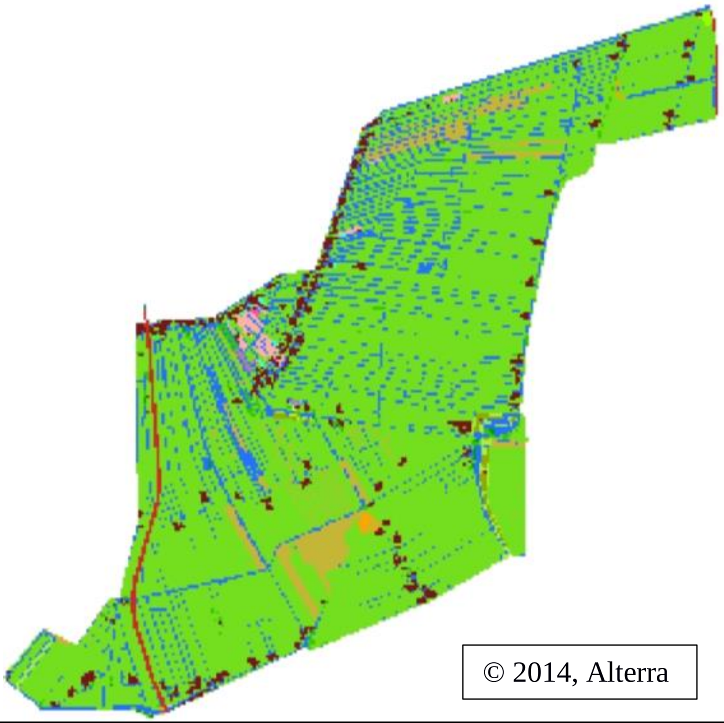

Table S2. Nature in the case study area (Stichting Ontwikkeling De Venen, 2010). Note that much of the 'realised new nature' is not yet detected and included in the ArcGIS data in Table S.1.1. above.

Old, new and planned nature	Current nature plan	Proposed in the interregional development plan 'Groene Ruggengraat'.
Pre-existing nature	69 ha	69 ha
Realised new nature	128 ha	128 ha
Total planned natural area	319 ha	387 ha
Remaining task	122 ha	190 ha

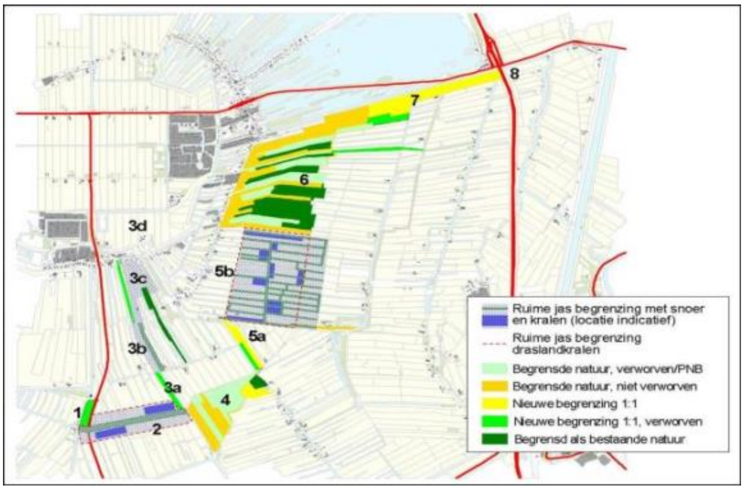

Figure S4. Focus areas for nature in the case study area (Stichting Ontwikkeling De Venen, 2010). Map indicates multiple categories: 'bestaande natuur' is pre-existing, 'verworven' is newly acquired, others are planned/potential.

Table S3. Agriculture in the case study area (Utrecht-West, 2012b).

Agrarian businesses	Number
Dairy (primary land user and main agricultural focus of the area management plans)	27
Other	22
Total	49
Involved in nature management	25
Involved in broadening their services and income base (multi-functional agriculture)	28

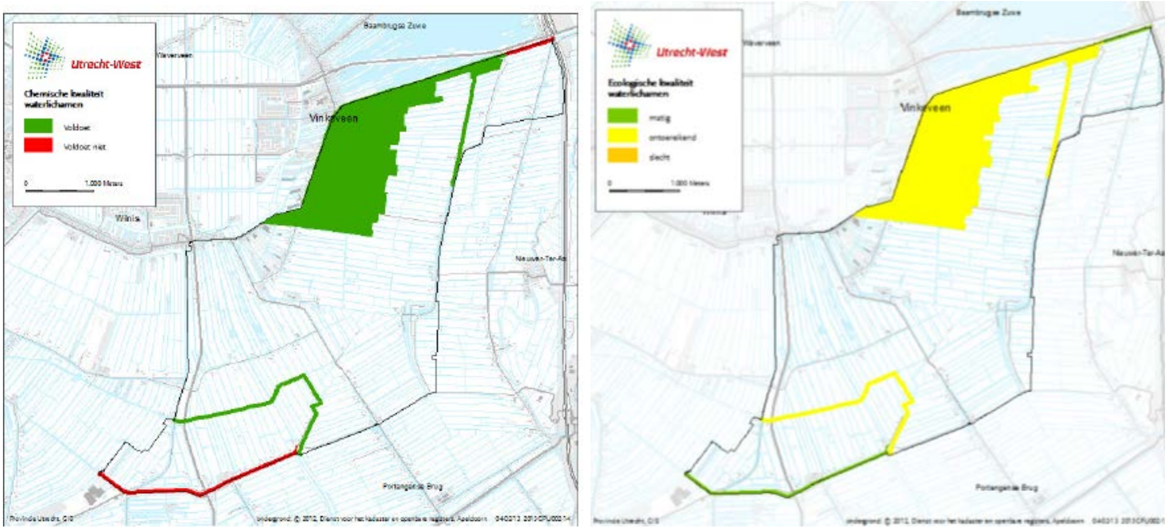

Figure S5. Water quality of bodies covered by the EU Water Framework Directive (Utrecht-West, 2012b). Left: chemical (green meets requirements, red doesn't). Right: ecological (green mediocre, yellow insufficient, orange poor).

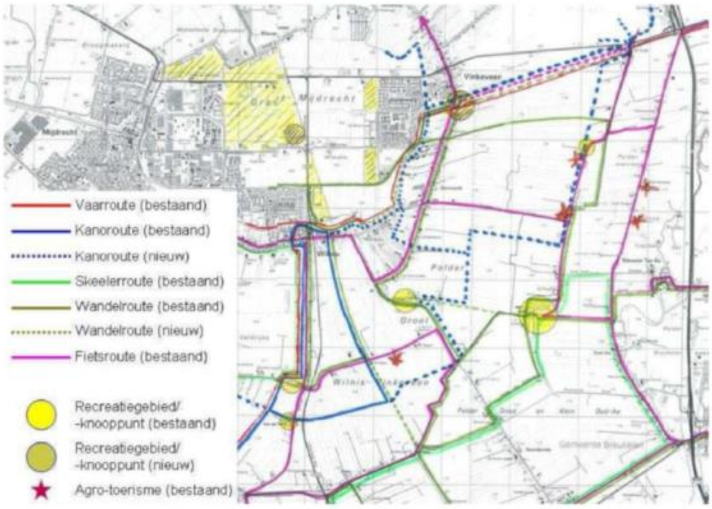

Figure S4. Planned recreation in the case study area (Utrecht-West, 2012b).

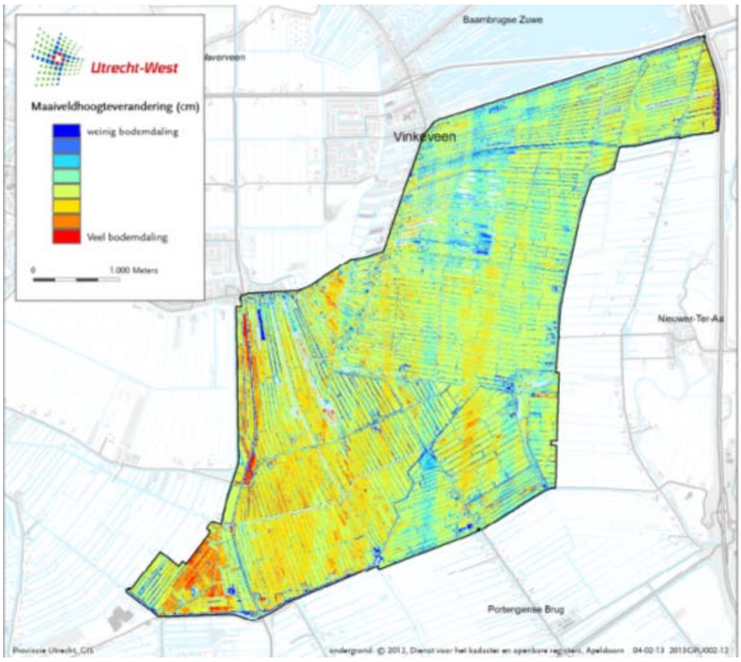

Figure S7. Soil subsidence in the case study area, 2005-2012 compared to 1990-2005 (Utrecht-West, 2012b). Blue is "little", red is "much" subsidence. Average subsidence in the area is 6 mm/yr, high-end is 12 mm/yr.

S2. Climate change impacts in the Venen-Vechtstreek

Climate change will have a number of effects in the Netherlands. The Dutch national climate scenarios (KNMI, 2014) assess these for four situations: a global temperature rise of 'moderate' (G) or 'warm' (W), entailing +1 or +2 °C in 2050, combined with either a low (subscript L) or high (subscript H) amount of change in the air circulation patterns over Europe. Effects are assessed on temperature, precipitation, sea level, wind & storm, visibility/fog, hail & thunderstorms, clouds, solar radiation, evaporation, humidity, and drought. Detailed spatial projections and impact studies are available as well (IPO, 2009; KNMI, 2009a). The case study is an area of wetlands (peat grassland), used for agriculture, nature, tourism, and water. It is particularly sensitive to: increasing temperatures (air, water), changes in precipitation (volume, frequency), evaporation and drought. Impacts can be expected in fields such as flood safety, water availability, water quality, ecosystems & biodiversity, agriculture, public health, and recreation & tourism (Van Minnen et al., 2013).

Climate change impacts can reduce water quality and lead to changes in water availability: decrease or increase in summer, increase in winter, increase in heavy precipitation (KNMI, 2014). Increased water temperatures can strongly impact the ecological status of the surface waters. Van Minnen et al. (2013) indicate a tipping point of 20-26 °C, beyond which the physiology and survival rates of animal species are affected. Heat and drought can also lead to more nutrients being released into surface water. Similarly, heavy precipitation can enhance nutrient leaching from fields into surface water. Eutrophication and heat can in turn increase microbial contaminations and algae blooms. Salinization could also increase: drought, affected by lower river discharge and sea level rise, leads to larger sea water intrusion inland and sea level rise may increase saline seepage in coastal areas. In the study area, salinization risk is relatively low, except in very warm summers when brackish, poor-quality water from outside the area will need to be used for maintaining the water levels (IPO, 2009; Verhoeven et al., 2012). Such events lead to a high risk of salinization, as well as to reduced water quality in general. Drought can also increase pollutant concentrations by reducing water volume.

Water temperature, availability, and quality influence the quality of nature in the study area, as noted above. Verhoeven et al. (2012) assessed the impacts in the study area (Table S4). Eutrophication poses a moderate risk to aquatic nature, but small risk to terrestrial nature. Drought risk is moderate in the scenario with unaltered air circulation patterns, and high in scenarios with changed circulation. Salinization low in most scenarios, but high in the scenario with changed circulation patterns, which can lead to very warm, dry summers. During such summers, brackish water may need to be imported from outside, as mentioned above. Changing climatic conditions will also influence the distribution of species (e.g. IPCC, 2014). Geographic zones where climatic conditions for species are favourable will shift, and will force species to search for a new habitat that fits their preferences. The speed of this shift is dependent on, among others, species characteristics and the availability and fragmentation of the area where species can move (Opdam and Wascher, 2004). Climate change can also influence bird migration patterns: warmer winters have already led to a decline in overwintering migratory birds from the north, and an increase in birds remaining in the country throughout the year. Changes in growing seasons may also change the timing of peak distributions of plants, insects, and animals. Ecological impacts are not yet clear, but it could lead to a mismatch in food availability (Van Minnen et al., 2013).

Table S4. Climate change impacts on nature in the study area (Verhoeven et al., 2012). Scenarios were based on KNMI (2009a,b): current situation, W: +2 °C in 2050 (similar to WL), WH: +2 °C in 2050 with circulation change (similar to W_H but with stronger summer drying), W+B: W+ plus optimized water management.

Impact	Risk/Vulnerability in 2050 per Scenario			
	Current	W	W+	W+B
Aquatic nature				
Eutrophication of ditches and canals	Moderate	Moderate	Moderate	Moderate
Salinization of ditches and canals	Small	Small	Large	Small
Terrestrial nature				
Eutrophication of new grassland nature	Small	Small	Small	Small
Drying of wet arid lands	Moderate	Moderate	Large	Large
Drying of humid meadowland	Moderate	Moderate	Moderate	Moderate
Drying of humid meadow bird grassland	Moderate	Moderate	Large	Large

Agriculture is affected through effects on precipitation, evaporation, and drought conditions. For the study area, this can increase the potential precipitation deficit during summer (KNMI, 2009a). Prolonged warm periods and dry spells reduce the amount of available water and lead to soil drying, impacting the quality of the grass. Heat and drought negatively impact dairy production. Reduced water quality will have implications for irrigation and agricultural production. Intense or prolonged rainfall reduces the accessibility and usability of agricultural lands. As noted above, drainage problems have been one of the main issues for agriculture in the area in the past. Wet periods and flooding could exacerbate the current tension in water level requirements for agriculture (low) and nature (high). There is relatively little crop production in the area. Consequently, various crop-related impacts of climate change are less relevant (cf. Van Minnen et al., 2013; KNMI, 2014). The longer growing season and increased CO_2 concentrations may have some beneficial effects due to higher grass yield.

Recreation may be affected by the impacts described above as well (cf. Van Minnen et al., 2013). Drought, heat, low water quality and levels, and potential negative effects on the landscape (via impacts on agriculture and nature) could be negative for recreation. Positive effects can also be expected, as the number of favourable days for outdoor recreation will increase, due to warmer weather, and residents from the surrounding Randstad cities may seek refreshment in the area during hot days.

Other relevant effects of climate change include those on soil subsidence, which can be exacerbated by drought. Public health will be affected via for instance heat and pollen/allergies. As a polder, the area is also flood-prone. Precipitation can lead to local water drainage-related flooding and riverine flood safety risks. Drought can also enhance flood risks in the study area, due to the presence of peat dikes and quays. Peat is greatly reduced in weight when it dries, reducing dike stability, and increasing the risk that it may shear. The town of Wilnis experienced such a dike breach during the European Heat Wave of 2003. The town was flooded and 1500 people were evacuated.

S3. Description of management options and resilience impacts in the Venen-Vechtstreek

S3.1. Agriculture

The measures to improve the agrarian function are aimed at upscaling and professionalising agrarian businesses and at water level management. Water level management has the purpose of protecting agricultural entrepreneurs from flooding and droughts, and of limiting soil subsidence.

In order to keep dairy farming economically healthy, the covenant suggests stimulating business upscaling. From a resilience point of view, this is not necessarily an improvement. Upscaling means less businesses for the same amount of cows, and therefore less redundancy. If one of those businesses has problems, a larger fraction of the economy is affected. The flux might increase if it is easier for a large company to mobilise resources for innovation or adaptation. Omnivory will also go down, as everything is more uniform within one company compared to several smaller companies, who may have different kinds of cows, food, suppliers, or processes.

Stimulating and supporting entrepreneurship by employing an area broker can improve the flux of information and knowledge. It can also keep the organisation of the area reasonably flat, if the task of the area broker is to connect people, skills and knowledge. However, if all communication has to go via the area broker, it adds a level of hierarchy, which decreases the flatness and can slow down communication. The area broker can provide knowledge and connections to stimulate agrarians to offer 'green and blue services', which offers co-benefits for recreation, nature and water quality.

Subsidising business plans aimed at making future business more sustainable and less specialised creates a synergy between green and blue economy, dairy farming and tourism. By providing green and blue services and agrotourism, additional to dairy farming, increases homeostasis. Businesses become less specialised, which increases omnivory.

Optimising plot arrangement and accessibility: by subsidising dams and culverts, the flux will go up as well as the redundancy. More water can be let out of the area because of better and more outlets. A farm that is better accessible allows faster movement of cattle in case of flooding.

Underwater drainage can be an effective way to stabilise the ground water level. The spread of dependence on water sources improves redundancy. It allows agriculture to handle higher water tables, which can have benefits for buffering, limits soil subsidence and is good for nature. A disadvantage is that more water input is required, potentially reducing water quality.

An additional pumping station will be built. This will improve flux and redundancy, and if it is used as part of a feedback system to keep the water level constant, it improves homeostasis.

S3.2. Recreation

The measures listed under recreation consist of making a canoe route through Donkereind, Demmerik and de Geuzensloot and hiking and biking routes through the nature reserves. This could attract nature lovers, which can be an incentive for policy makers to consider the implications on nature whenever they take decisions about the area. More tourists will increase the income of farmers offering opportunities for agrotourism, like accommodation for tourists. The measures for recreation will increase homeostasis of the combined categories nature, agriculture and recreation.

Any of the measures suggested for nature can also have beneficial effects for recreation because they improve the attractiveness of the area. More diverse nature means more chances for recreation. However, recreation can also put pressure on nature.

S3.3. Nature

The measures that are proposed for nature aim to provide a safe habitat for species, either by cleaning water or keeping dirty water out of the area, or by creating a diverse landscape. They also aim to help species spread across the area or to or from other areas.

Reducing water inlet can be good for water quality, by keeping polluted water out of the area. It reduces redundancy, unless the inlet of water is kept flexible, and can be raised in dry periods. Minimising the inlet of water will decrease the flux, which keeps contaminants in the area for longer.

Realisation of balanced fertilisation in agriculture improves omnivory, making the ditches suitable to more species. Biodiversity allows for feedback loops, therefore improving homeostasis.

More ecological/organic farming will increase biodiversity and could have many co-benefits, such as cleaner water and air, nicer recreation, or reduced public health risks. It also benefits agriculture by adding a new type of farming to traditional farming practice, increasing omnivory.

Dredging secondary and tertiary waterways to a depth of 0.5m, increases diversity of aquatic macroinvertebrates. For recreation, it could also add to omnivory, by increasing possibilities to access the area by canoe.

For the conservation of rare species, new marshland nature will be realised on the lowest plots. These plots can provide water buffering. Less polluted water will have to be pumped into higher nature areas in order to keep them wet, improving water quality, benefiting nature and agriculture. Moving farmers from these locations will however take considerable effort, time, and money. It will reduce need for drainage in agricultural areas, which will decrease soil subsidence.

In the core area for meadow birds, a marshland connection will be realised, employing a system of capillaries, natural banks and marshland 'beads' (small patches of marshland). This may give some species more ways to travel from one area to the next, increasing omnivory. The system will also function as an extra 'filter', improving water quality and increasing omnivory. For landscape reasons, the connection will be split up into two different routes (Table 2). This may increase omnivory by introducing more ways for some species to move through the area. However, the individual corridors will be less wide, potentially increasing their vulnerability. Marshland and wetland increase water quality. They are also beneficial for recreation (landscape variation, birds) and for water retention, which buffers against drought and flooding (benefiting nature, agriculture).

S3.4. Clean water

The measures for clean water should improve the suitability of the area as a reservoir of drinking water. These measures are not aimed at improving nature, but have co-benefits for both nature and recreation. Other measures to improve water quality are under nature or agriculture.

The realisation of 35 km of natural banks will clean nutrients out of the water. This measure has co-benefits for both nature and recreation when this new type of banks (increasing omnivory) provides a habitat for various plant and animal species.

References

Hazeu, G.W., C. Schuiling, G.J. Dorland, G.J. Roerink, H.S.D. Naeff en R.A. Smidt (2014). Landelijk Grondgebruiksbestand Nederland, versie 7 (LGN7): Vervaardiging, nauwkeurigheid en gebruik. Alterra, Wageningen University & Research Centre, Wageningen.

IPCC (2014) Climate change 2014: Impacts, adaptation and vulnerability. Cambridge University Press, Cambridge.

IPO (2009) Klimaateffectatlas: Inspelen op klimaatverandering. Association of Provinces of the Netherlands (IPO), The Hague.

KNMI (2009) Klimaatschetsboek Nederland: Het huidige en toekomstige klimaat. Royal Netherlands Meteorological Institute (KNMI), De Bilt.

KNMI (2014) KNMI’14 climate scenarios for the Netherlands. Royal Netherlands Meteorological Institute (KNMI), De Bilt.

Opdam P, Wascher D (2004) Climate change meets habitat fragmentation: Linking landscape and biogeographical scale levels in research and conservation. *Biol Conserv* 117:285-297.

Stichting Ontwikkeling De Venen (2010) Gebiedsconvenant Groot Wilnis-Vinkeveen 2010-2020. Stichting Ontwikkeling De Venen, Utrecht.

Utrecht-West (2012a). Begrenzing gebiedsproject Groot Wilnis-Vinkeveen. Province of Utrecht, Utrecht. http://www.utrecht-west.com/Projecten/Groot+Wilnis+Vinkeveen/Documenten+Groot+Wilnis+Vinkeveen/HandlerDownloadFiles.ashx?idnv=435009

Utrecht-West (2012b). Gecombineerde voortgangsrapportage & effectmonitoring Groot Wilnis-Vinkeveen. Programmabureau Utrecht-West, Province of Utrecht, Utrecht. http://utrecht-west.mett.nl/Projecten/Groot+Wilnis+Vinkeveen/Documenten+Groot+Wilnis+Vinkeveen/HandlerDownloadFiles.ashx?idnv=435008

Van Minnen J, Ligtvoet W, et al. (2013) The effects of climate change in the Netherlands: 2012. PBL Netherlands Environmental Assessment Agency, The Hague.

Verhoeven J, Paulissen M, et al. (2012) Klimaateffecten op de Natura 2000 moerascorridor: Quick scan in het Groene Hart. Province of Zuid-Holland, The Hague.
